# The Intracellular Loop of the Glycine Receptor: It’s not all about the Size

**DOI:** 10.3389/fnmol.2016.00041

**Published:** 2016-06-03

**Authors:** Georg Langlhofer, Carmen Villmann

**Affiliations:** Institute of Clinical Neurobiology, University of WürzburgWürzburg, Germany

**Keywords:** GlyR receptors, synaptic inhibition, intracellular domain, interaction partners, posttranslational modifications

## Abstract

The family of Cys-loop receptors (CLRs) shares a high degree of homology and sequence identity. The overall structural elements are highly conserved with a large extracellular domain (ECD) harboring an α-helix and 10 β-sheets. Following the ECD, four transmembrane domains (TMD) are connected by intracellular and extracellular loop structures. Except the TM3–4 loop, their length comprises 7–14 residues. The TM3–4 loop forms the largest part of the intracellular domain (ICD) and exhibits the most variable region between all CLRs. The ICD is defined by the TM3–4 loop together with the TM1–2 loop preceding the ion channel pore. During the last decade, crystallization approaches were successful for some members of the CLR family. To allow crystallization, the intracellular loop was in most structures replaced by a short linker present in prokaryotic CLRs. Therefore, no structural information about the large TM3–4 loop of CLRs including the glycine receptors (GlyRs) is available except for some basic stretches close to TM3 and TM4. The intracellular loop has been intensively studied with regard to functional aspects including desensitization, modulation of channel physiology by pharmacological substances, posttranslational modifications, and motifs important for trafficking. Furthermore, the ICD interacts with scaffold proteins enabling inhibitory synapse formation. This review focuses on attempts to define structural and functional elements within the ICD of GlyRs discussed with the background of protein-protein interactions and functional channel formation in the absence of the TM3–4 loop.

## Introduction

Glycine receptors (GlyRs) are the major inhibitory neurotransmitter receptors in adult spinal cord and brainstem. They are important for motor coordination and respiratory rhythm. Disturbances in glycinergic neurotransmission by: (i) mutated genes encoding various GlyR subunits or adjacent proteins of the glycinergic receptor complex; (ii) receptor editing or; (iii) receptor modulation by posttranslational mechanisms lead to neuromotor deficits (hyperekplexia), pain sensitization and autism spectrum disorders (Lynch, 2004; Schaefer et al., 2013; Bode and Lynch, 2014; Pilorge et al., 2015).

GlyRs are members of the superfamily of Cys-loop receptors (CLRs) such as nicotinic acetylcholine receptors (nAChR), 5HT_3_ receptors, and GABA_A/C_ receptors. They all share a common disulfide bridge in the extracellular N-terminal domain between conserved cysteine residues. GlyRs are pentameric receptors composed of 2α and 3β subunits (Grudzinska et al., 2005). Four different α subunits and one β subunit are known. Functional diversity is enhanced by alternative splicing processes, which has been described for all subunits (Kuhse et al., 1991; Malosio et al., 1991; Nikolic et al., 1998; Oertel et al., 2007; Hirata et al., 2013).

**Figure 1:**
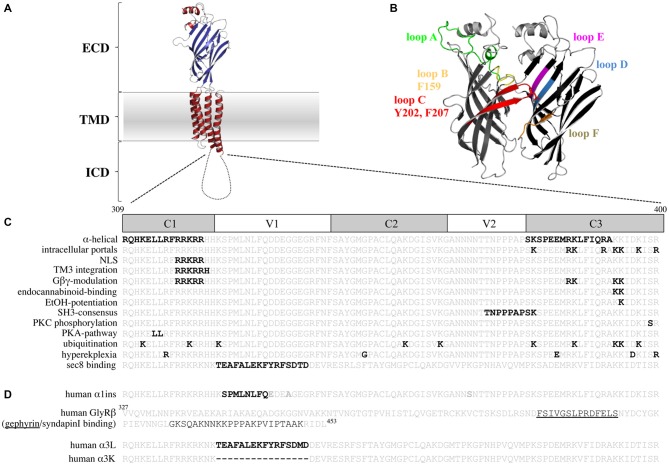
**The glycine receptor (GlyR) intracellular domain (ICD)—important residues and domains. (A)** Model of a GlyR monomer based on the x-ray structure of *Danio rerio* GlyRα1 (Du et al., 2015) with the extracellular domain (ECD), the transmembrane domain (TMD), and the ICD. Note, the ICD is drawn as a cartoon due to lack of structural information. **(B)** Model of a GlyR ECD dimer interface. Loop structures contributing to glycine binding are shown. Principal subunit (+; left): loop A (green), loop B (yellow, with F159), loop C (red, with Y202 and F207); complementary subunit (−; right): loop D (blue), loop E (magenta), loop F (brown). **(C)** TM3–4 loop sequences of the human GlyRα1 (residues 309–400) and human α3 (last line) are shown. Constant (C) and variable (V) regions of the TM3–4 loop are marked. Bold black letters—all residues that have been functionally investigated *in vitro* (structurally and functionally important residues for ion permeation and desensitization, residues that bind intracellular proteins, residues involved in receptor trafficking and TM3 integration, residues that bind drugs and Gβγ proteins, posttranslational modifications, residues affected in human patients). **(D)** TM3–4 loop sequences of the human α1 (α1ins) and α3 splice variants (long-α3L and short-α3K) are shown. Splice inserts are marked with black bold letters. In the GlyRβ TM3–4 loop sequence binding sites for gephyrin (underlined) and syndapin are marked. Note, the β TM3–4 loop is longer (residues 327–453) compared to α1 (309–400) and α3 (α3L 309–400 and α3K 309–385).

Most of the knowledge about GlyR signal processing comes from *in vitro* mutagenesis studies on structure-function relationships. Recently the x-ray structure of GlyRα3 and the cryo-electron microscopic structure of α1 were solved (Du et al., 2015; Huang et al., 2015). These structures provided deeper insights into the mechanisms of signal processing and gating. Interestingly, x-ray crystallography of CLR members was only possible when the large intracellular loop between TM3–4 was replaced by a short peptide. The TM3–4 loop harbors the highest variability among all CLRs in terms of length and sequence variations. These loop structures mediate subfamily-specific interactions with intracellular binding partners (Goyal et al., 2011). In GlyRs, the TM3–4 loops interact with the scaffold protein gephyrin important for synaptic anchoring or signal transduction processes. In addition, the TM3–4 loop is modified by posttranslational modifications and binds allosteric modulators that in turn influence functional ion channel properties (Figures 1A–D; Ruiz-Gómez et al., 1991; Kirsch and Betz, 1995; Yevenes et al., 2008; Yevenes and Zeilhofer, 2011). Subdomains of the GlyR TM3–4 loop have been demonstrated to be important for receptor trafficking to the cellular membrane and the nucleus (Sadtler et al., 2003; Melzer et al., 2010).

## Importance of Glycine Receptors for Inhibitory Neurotransmission

In the nerve muscle circuit, GlyRs control excited motoneurons in spinal cord and brainstem. Motoneuron activation is enabled by released glutamate from dorsal root ganglia. In turn, activated motoneurons fire action potentials towards the neuromuscular endplate where the signal is transmitted via acetylcholine to propagate along muscle fibers resulting in muscle contraction. To balance motoneuron firing, inhibitory GlyRs localized within the motoneuronal membrane are activated by release of glycine from neighboring interneurons. These interneurons are excited by collateral axons of the motoneurons. As a consequence, motoneurons are hyperpolarized and excitation is dampened. This feedback control by GlyRs restores the balance between excitation and inhibition (Schaefer et al., 2012). Using similar mechanisms, GlyRs mediate respiratory rhythms in PreBöt (pre-Bötzinger complex) nuclei of the brainstem (Winter et al., 2009; Janczewski et al., 2013). An impaired glycinergic inhibition in the brainstem of the mouse mutant *oscillator* leads to decreased breathing frequency caused by prolongation of expiratory duration. This results in death of affected mice around postnatal day 21 due to respiratory acidosis (Markstahler et al., 2002). Minor GlyR expression has been determined in the retina, inner ear, and the hippocampus (Harvey et al., 2004; Heinze et al., 2007; Dlugaiczyk et al., 2008; Lynch, 2009; Aroeira et al., 2011).

In the hippocampus, GlyRs are mainly found at extrasynaptic sites pointing to a function in tonic activation processes (Aroeira et al., 2011). These extrasynaptic receptors are formed by homomeric α2 and α3 GlyR subunits. A gain of function GlyRα3 variant (α3^P185L^) was previously identified in human hippocampectomies from patients with temporal lobe epilepsy (Meier et al., 2005; Eichler et al., 2008). Additionally, the hippocampus of patients with epilepsy expresses predominantly the long splice isoform of α3 (α3L; Eichler et al., 2009). Both findings were used to generate a mouse model with neuron-type specific expressions of the GlyRα3L^P185L^ to study homeostatic effects that control synaptic neurotransmission. The estimated presynaptic expression of GlyRα3^P185L^ in glutamatergic terminals facilitated neurotransmitter release (Winkelmann et al., 2014). As a consequence, enhanced hyperexcitability leads to recurrent epileptoform discharge impairing cognitive function and discriminative associative memory (Winkelmann et al., 2014). Changes in cognitive function and discriminative associative memory have been analyzed with the reward-based 8-arm radial maze test that discriminates between working memory (number of entries into an arm that was never baited) and reference memory (re-entries into an arm visited in the ongoing trail).

In contrast, specific expression of GlyRα3L^P185L^ in parvalbumin-positive interneurons generated hypoexcitability and triggered anxiety-like behavior (Winkelmann et al., 2014). Increased anxiety of GlyRα3L^P185L^ mice was verified by a preference for the dark using the dark/light test, decreased entries into the center in an open field, and less time spent and decreased numbers of entries into the open arms using the elevated plus maze test (Winkelmann et al., 2014). In conclusion, increased presynaptic function represents a pathogenic mechanism able to alter neural network homeostasis and thereby control neuronal network excitability and trigger neuropsychiatric symptoms (Winkelmann et al., 2014).

Inhibition of postsynaptic GlyRα3 by PGE2- (prostagladin E2) induced phosphorylation underlies central inflammatory pain sensitization. This process depends on the activation of protein kinase A that phosphorylates α3 at residue S346 localized in the TM3–4 loop (Harvey et al., 2004). These findings initiated a series of pharmacological studies with GlyRα3 as a promising target in pain therapy (Lynch and Callister, 2006).

The involvement of GlyRs in autism spectrum disorders is based on genetic findings and knockout mice although the molecular mechanisms behind their involvement in the excitation/inhibition imbalances are not completely understood (Tabuchi et al., 2007; Pilorge et al., 2015). The analysis of a rare human X-linked *GLRA2* microdeletion (deletion of exons 8 and 9 that refer to the TM3–4 loop) associated with autism exhibited lack of surface GlyR expression *in vitro* and severe axon-branching defects in zebrafish (Pilorge et al., 2015). A knockout of *Glra2* in mice revealed deficits in object recognition memory and impaired long-term potentiation in the prefrontal cortex. In summary, these data provide evidence for a link of altered glycinergic inhibition to social and cognitive impairments (Pilorge et al., 2015).

The role of GlyRs detected in non-neuronal tissues, e.g., immune cells, endothelial cells, hepatocytes, renal cells is not completely understood but argues for other functions than a neuronal ion channel (Van den Eynden et al., 2009).

## Human and Murine Mutations Found in GlyRα1 Intracellular Domain (ICD)

GlyR mutations can result in the neuromotor disorder hyperekplexia. The most common cause for hyperekplexia are mutations in the *GLRA1* gene which was mapped to the disease in 1993 (Shiang et al., 1993). The second most common cause for hyperekplexia results from mutations in the *SLC6A5* gene encoding the presynaptic glycine transporter 2 (GlyT2; Rees et al., 2006). Mutant GlyT2 variants represent the presynaptic component of the disease. Rare forms of the disease are generated by mutations in genes encoding other postsynaptic proteins of the inhibitory synapse, e.g., gephyrin and collybistin (CB).

GlyRα1 mutations are distributed over the entire sequence. Among these, most of the dominant inherited mutations are localized in the ion channel domain (TM2) and adjacent loop structures. These mutants are accompanied by functional deficits such as lower maximal currents, reduced single channel conductance, enhanced desensitization or decreased ligand-binding efficacy (Saul et al., 1999; Becker et al., 2008; Chung et al., 2010). In contrast, recessive mutants influence receptor biogenesis, trafficking, and receptor stability (Villmann et al., 2009b; Schaefer et al., 2015).

So far, only five human mutations, R316X, G342S, E375X, D388A, and R392H have been identified in the GlyRα1 TM3–4 loop (Figure 1C). Three of them (R316X, D388A, R392H) are compound heterozygous. Compound heterozygosity refers to two recessive alleles (W68C/R316X, L291P/D388A, and R252H/R392H) that result in hyperekplexia in a heterozygous state (Vergouwe et al., 1999; Rees et al., 2001; Tsai et al., 2004; Chung et al., 2010; Bode and Lynch, 2013). *In vitro* studies on R392H revealed decreased inward currents, reduced expression and less stability as the underlying pathological mechanism. These effects were more pronounced when R392H was coexpressed with R252H. Receptors composed of R252H and R392H were non-functional, arguing for a dominant effect of R252H localized in close proximity to the ion channel pore (Villmann et al., 2009b).

GlyRα1 variants R316X and E375X lead to truncated α1 subunits. Truncations of receptor proteins result in significantly decreased surface expression due to protein misfolding and abnormal receptor trafficking (Villmann et al., 2009a; Kang et al., 2015; Schaefer et al., 2015). As a consequence, insufficient receptor densities lead to deficiency of functional ion channels.

A similar TM3–4 loop truncation of the closely related GABA_A_R γ2 subunit is associated with generalized epilepsy with febrile seizures plus (GEFS+; Kang et al., 2015).

**Figure 2:**
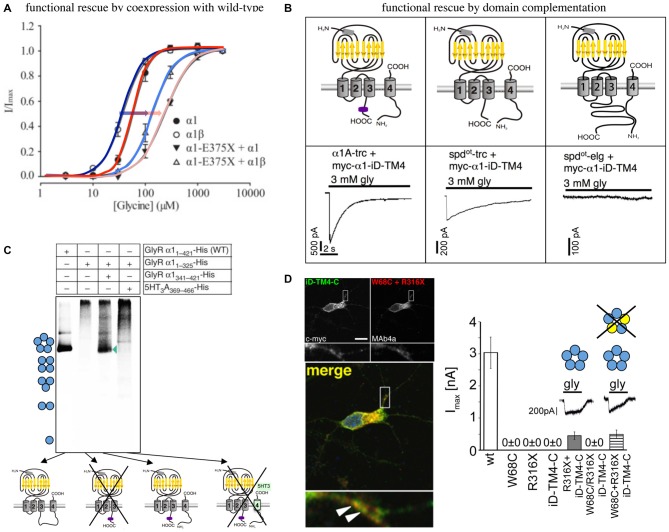
**Functional rescue of truncated non-functional GlyRα1 variants. (A)** Functional rescue of the human truncated variant E375X by wild-type (wt) α1 or α1β. Note, the shift in EC_50_ upon coexpresion with wt GlyRs (shift from dark blue to light blue with coexpressed α1β, red to pink with coexpressed α1, also marked by arrows), modified from Bode et al. (2013). **(B)** Principle of domain complementation of the α1 splice variants generated in the mouse mutant *oscillator* (spd^ot^-trc—truncated form, spd^ot^elg—long splice variant, α1-trc—corresponding wt truncation) together with the complementation domain (myc-α1-iD-TM4). The latter is composed of most of the α1 TM3–4 loop sequence, TM4, and the C-terminus (upper cartoons). Maximal current amplitudes of coexpressed truncated or elongated α1 together with the complementation construct (lower current traces). Truncated α1 wt and spd^ot^-trc differ in rescue efficacy, the long *oscillator* variant was not rescued most probably due to steric hindrance between both GlyR domains essential for functional complementation (see upper right cartoon), modified from Villmann et al. (2009a). **(C)** Domain coexpression similar to **(B)**. The formation of pentamers depends on the presence of the complementation construct harboring most of the TM3–4 loop, TM4, and the C-terminus. If the complementary domain is from another CLR, pentamers were not formed (marked by a cross), modified from Haeger et al. (2010). **(D)** Human W68C and R316X variants (refer to compound heterozygous mutations of a patient) coexpressed with complementation construct in hippocampal neurons (dendritic colocalization, left images). Summarized functional analysis of domain complementation (right diagram), modified from Schaefer et al. (2015).

An *in vitro* analysis of α1 E375X revealed no surface expression of the truncated α1 protein when expressed alone to form homomeric receptor complexes. Coexpression of α1E375X with wild-type (wt) α1 or α1β led to functional ion channel formation. The observed current amplitudes were smaller and EC_50_ values were increased for GlyRs formed by α1wt/α1E375X/β in comparison to homomeric α1 and heteromeric α1β wt (Figure 2A). This simulation of the *in vivo* configuration constitutes the potential of E375X to integrate into pentamers, its transport to the cell surface and finally its impact on GlyR function (Bode and Lynch, 2013). Similar effects have been observed for the GlyRα1 ICD variant D388A. Mutant α1D388A receptors were only recruited to the cellular membrane in presence of either α1 or α1β wt (Bode et al., 2013).

R316X showed impaired trafficking with a small fraction of mutated GlyRs expressed at the cellular surface but insufficient to generate functional ion channels (Schaefer et al., 2015).

A TM3-4 loop truncation in the mouse mutant *oscillator* results in absence of truncated protein from the organism. *Oscillator* carries a 7 bp deletion and depending on the use of an alternative splice acceptor site generates two different transcripts although neither is translated into α1 protein *in vivo* (Kling et al., 1997). Lack of translation of both transcripts induces severe neuromotor deficits in homozygous *oscillator* mice starting at postnatal day 14. These deficits increase progressively until death at postnatal day 21. During this period GlyRs undergo a subunit switch from homomeric α2 (embryonic isoform) to heteromeric adult GlyRs (α1β, α3β). Obviously, there is no compensation by other GlyRs to the lack of functional α1β receptors in homozygous *oscillator* mice (Buckwalter et al., 1994; Kling et al., 1997). Thus, *oscillator* represents a GlyR NULL mutation.

An *in vitro* coexpression of the truncated *oscillator* GlyRα1 protein (*spd*^ot^-trc) together with a complementary truncated wild-type α1 construct (harboring most of the TM3–4 loop sequence, TM4, and the C-terminus = myc-α1-iD-TM4; Figure 2B) restored surface expression of both GlyR domains arguing for lack of precise quality control in the overexpression system (Villmann et al., 2009a). The coexpression of the non-functional truncated GlyRα1 isoform (*spd*^ot^-trc) together with the lacking protein portion (myc-α1-iD-TM4) on a separate plasmid in the same cell regenerated ion channel functionality (GlyRα1 rescue = functional complementation of an ion channel from for themselves non-functional ion channel domains). These findings suggest that GlyRs are composed of independent folding domains able to interact with each other to complement channel functionality (Figure 2B; Villmann et al., 2009a). Using similar GlyR N- and C-terminal domains, it was further shown that non-functionality of truncated GlyRs lacking the TM3–4 loop, TM4 and the C-terminus is due to the inability to form pentameric receptor complexes (Figure 2C; Haeger et al., 2010).

How do these independent folding domains interact? An interaction between differently charged residues was analyzed by stepwise truncation of the complementation construct from its N- to the C-terminus. A lack of more than 55 residues from the TM3–4 loop resulted in non-functionality. Interestingly, the coexpression of three GlyR domains regenerated functionality at least to some extent further supporting the finding for independent folding domains of the GlyR (Unterer et al., 2012).

An application of the domain complementation approach to truncated human variants yielded similar results. The human α1 variant R316X was coexpressed with a corresponding C-terminal complementation construct (iD-TM4-C). The functional restoration of the respective GlyRs achieved 20% of ion channel efficacy compared to the wild-type situation. R316X was identified in a patient concomitant to W68C. The mutant W68C significantly decreased receptor trafficking to the cellular surface. A coexpression of W68C, the complementation construct, and R316X generated functional ion channels indistinguishable from GlyRs lacking W68C (Figure 2D). Therefore, it was concluded that the mutant W68C in the extracellular domain (ECD) does not hinder R316X from forward trafficking and integration into the pentameric arrangement (Schaefer et al., 2015).

Hence, GlyRs are able to assemble from independent folding domains and generate functional ion channels. This process does not require the integrity of the GlyR ICD rather subdomain interactions may mediate the efficacy of GlyR ion channel functionality.

In addition to the TM3–4 loop, the ICD also comprises the short intracellular loop connecting TM1 and TM2. The role of the TM1–2 loop in hyperekplexia has been defined by functional studies of the mutant P250T (Saul et al., 1999). Residue P250 is localized in very close proximity to the inner vestibule of the ion channel. The introduction of a threonine at position 250 leads to fast-desensitizing receptors with decreased glycine sensitivity. A mutagenesis series of residue 250 determined side volume and hydropathy as important mediators in the pathology underlying P250T (Breitinger et al., 2001).

## Glycine Receptor Structure

Since 2011, the x-ray structures of several CLR members have been solved. These structures together with electron cryo-microscopy structures revolutionized our current knowledge about conformational rearrangements of the ion channel in the presence of agonists and antagonists leading to open and closed channel conformations (Unwin, 2005; Hassaine et al., 2014; Miller and Aricescu, 2014; Du et al., 2015). A closer view onto the CLR structure revealed an architecture of two domains: the ECD able to bind the ligand and the transmembrane domain (TMD) encompassing four α-helical transmembrane segments, connected by intra- or extracellular loop structures (Figure 1A). The crystal structures of the large intracellular loops of the GABA_A_ receptors, the 5HT_3_ receptors, and the GlyRs between transmembrane segments 3 and 4 have not been solved yet most probably due to hindrance of crystal formation when present.

The recently solved structures of GlyRα1 and GlyRα3 provided novel insights into GlyR functioning. Conformational rearrangements involve specific loop structures of the ECD as well as the ECD-TMD interface. These rearrangements enable ion channel gating as a consequence of an anti-clockwise outward rotation of TMD during opening of the ion channel pore. A prerequisite for glycinergic signal transduction is agonist-binding to the ligand-binding pocket formed by residues of loops A-F (Figures 1A,B). Ligand-binding is stabilized by aromatic residues e.g., F159, Y202, F207 within the pocket. Following binding, the signal is transmitted via extensive interactions near the ECD-TMD interface including the β1–2 loop, the Cys loop, and the M2–M3 loop at the principal side of the ligand-binding interface with loops β1–2, β8–9 and pre-M1/M1 of the complementary side (-) of the pocket (Du et al., 2015). Due to flexibility of loops C and β8–9, these loop structures initiate the rearrangement of the conformation from the open into the closed form by a backward movement involving the same loop structures and domains (Du et al., 2015). From crystallographic analysis there are so far no hints for an involvement of the intracellular loop between TM3–4 in signal transduction processes due to lack of its presence in constructs used for x-ray crystallography. Voltage-clamp fluorometry experiments however provided evidence for the participation of the TM3–4 loop structure in the rearrangement of M3 and M4 during ion channel opening. In this context it was demonstrated that M3 and M4 undergo large transitions compared to M1 and M2 movements (Han et al., 2013a).

## Structural Determinants of the GlyR ICD

In contrast to eukaryotic CLRs (nAChRs, GABA_A/C_Rs, GlyRs, and the 5HT_3_ receptors), the prokaryotic CLR-homologs ELIC (*Erwinia chrysanthemi* *ligand-gated ion channel*) and GLIC (*Gloeobacter violaceus ligand-gated ion channel*) carry very short intracellular loop structures (Hilf and Dutzler, 2008; Nury et al., 2011).

Chimeric CLRs (5HT_3A_-GLIC, GlyR-GLIC) harboring mainly the short heptapeptide SQPARAA (TM3–4 loop of GLIC) instead of their receptor-specific TM3–4 loop were able to form functional ion channels, which differ in single channel conductances and desensitization compared to wild-type receptors. Their overall properties, such as ion selectivity, efficiency of ligand-binding and current amplitudes were unaffected (Jansen et al., 2008; Papke and Grosman, 2014; Moraga-Cid et al., 2015). Thus, the amino acid sequence of the TM3–4 loop determines subclass-specific ion channel properties. All studies concerning chimeric receptors have been performed in overexpression systems *in vitro* leaving the question for an *in vivo* effect of chimeric proteins unanswered.

Our structural knowledge of the TM3–4 loop is limited to small segments close to TM3 and TM4. The rest of the TM3–4 loop seems to be disordered (Unwin, 2005). The C-terminal end of the TM3–4 loop of cation-selective CLRs forms an α-helical domain, called the MA stretch (membrane-associated stretch; Unwin, 2005; Hassaine et al., 2014). A large content of charged residues within the MA stretch face a lateral tunnel or portal. These portals enable the permeation of the incoming ions and influence ion channel conductance of the appropriate channel (Kelley et al., 2003).

**Figure 3:**
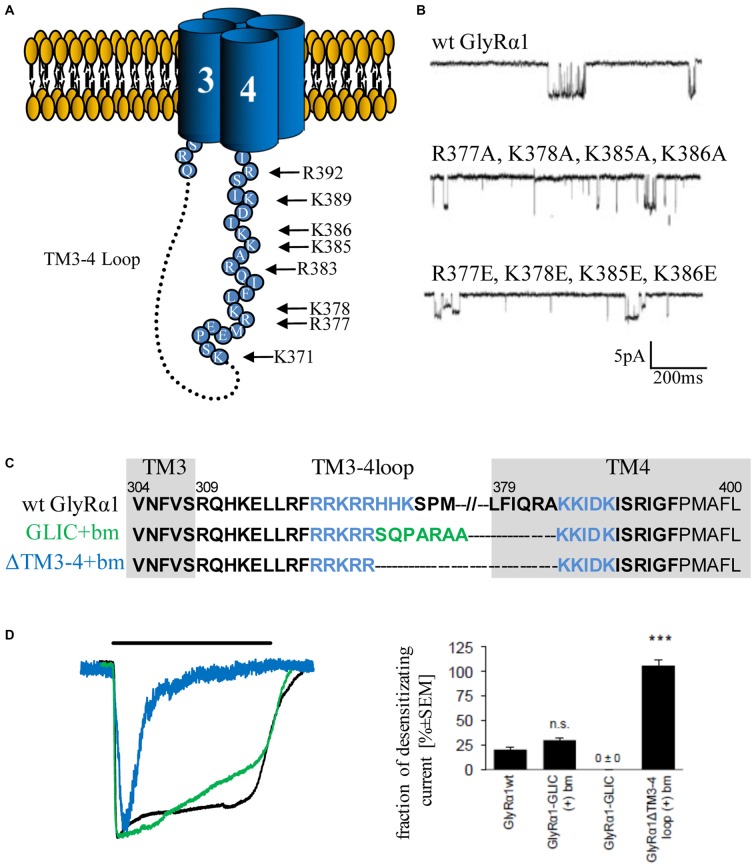
**Single channel conductance and desensitization are determined by intracellular portals and TM3–4 loop length. (A)** Model of intracellular portals formed by positively charged residues within the membrane-associated (MA) stretch of the TM3–4 loop of the GlyRα1 (modified from Carland et al., 2009). **(B)** Mutations of portal forming residues (quadruple mutations α1^R377A/K378A/K385A/K386A^ and α1^R377E/K378E/K385E/K386E^ have been investigated in comparison to wt) result in reduction of single channel conductance (Carland et al., 2009). **(C)** Truncated GlyRα1 used to investigate the influence of the loop length on receptor desensitization (Langlhofer et al., 2015). The sequence between the basic motifs (blue, bm) has been deleted (ΔTM3–4(+)bm) and replaced by the short *Gloeobacter violaceus ligand-gated ion channel* (GLIC) loop SQPARAA (green, GLIC(+)bm). **(D)** The connection of both basic motifs (blue) resulted in very fast desensitizing GlyRs compared to wt (black). Insertion of the GLIC-loop between the basic motifs had no influence on desensitization (modified from Langlhofer et al., 2015). n.s., not significant. Level of significance, ***p < 0.001.

The structure of the serotonin receptor provided some hints that there is a second α-helical stretch at the beginning of the TM3–4 loop (Figures 1C, 3A). The formation of intracellular portals is allocated by the C-terminal MA-stretch and obstructed by the N-terminal helix called MX-helix in a presumably closed channel conformation (Hassaine et al., 2014). The existence of such portals in GlyRs has been proposed due to sequence homology (Carland et al., 2009). Mutations of eight basic residues within the supposed glycinergic portals resulted in non-functional receptors. Moreover, quadruple mutations of positively charged residues (α1^R377A/K378A/K385A/K386A^ and α1^R377E/K378E/K385E/K386E^) reduced ion channel conductance at negative membrane potentials (Figure 3). Therefore, these portals are indeed features of an extended glycine receptor permeation pathway (Figures 1C, 3A,B). The positive charges surrounding the intracellular portals are assumed to electrostatically attract incoming anions to the intracellular compartment (Carland et al., 2009). CD spectroscopy further revealed the existence of α-helical elements close to TM3 and TM4 in GlyRα1 (Burgos et al., 2015).

The TM3–4 sequence of GlyRs can be subdivided into variable and conserved regions (Melzer et al., 2010; Figure 1C). Basic stretches are highly conserved among various GlyRs. Two other motifs have been determined to the variable region, a poly “NNNN” motif and a proline-rich stretch present in α and β subunits. The role of the asparagine-rich subdomain is completely unsolved.

The existence of a poly-proline helix type II (PPII) within the TM3–4 loop of the GlyR formed by the poly-proline stretch has been proposed by CD-spectroscopy (Cascio et al., 2001; Breitinger et al., 2004). PPII helices are helical secondary structures with a perfect 3-fold rotation symmetry forming SH3 consensus sequences (*SRC homology 3 domain consensus sequences*, Rath et al., 2005). The recognition motif for the PPII helix xxPxxP is highly conserved among all GlyR subunits and is involved in binding of intracellular partners to the GlyRβ loop (Figure 1D; Koch et al., 2011; Del Pino et al., 2014). Syndapin was identified as a binding partner of the ^384^KxxPxxPxxP^394^ motif in GlyRβ. The interaction between syndapin I and GlyRβ was greatly diminished when the second proline was exchanged by another residue (Del Pino et al., 2014). A miRNA knockdown of syndapin I in cultured primary spinal cord neurons assigned syndapin I as a mediator in GlyR trafficking or even anchoring (Del Pino et al., 2014). The latter needs further investigations to be proven.

Neuroligin 2 or the GABA_A_ receptors α2 harbor proline-rich sequences similar to the ^365^PPPAP^369^ motif in GlyRα1 and ^385^PPPAKP^390^ GlyRβ subunits. The interactions of these proline-rich stretches of neuroligin 2 or GABA_A_R α2 with the SH3 domain of CB underlie a novel regulatory mechanism for formation and function of inhibitory postsynapses (Soykan et al., 2014). CB has, however, never been shown to directly interact with GlyRs.

A further intracellular protein interaction has been attributed to the 15 residues splice cassette of GlyRα3L in the TM3–4 loop. GlyRα3L binding to the vesicular trafficking protein Sec8 targets GlyRα3L to presynaptic sites. Colocalization with the vesicular presynaptic marker VGLUT1 confirmed axonal trafficking of GlyRα3L towards presynaptic terminals (Winkelmann et al., 2014).

In conclusion, emerging evidences suggest a so far underestimated role of the GlyR TM3–4 loop in the interaction with other intracellular proteins beside gephyrin connecting the receptor to cytoskeletal elements, regulating receptor trafficking and synaptic localization.

## Motifs Important for Trafficking and Modulation of Channel Physiology by Pharmacological Substances

Basic residues ^316^RFRRKRR^322^ localized within the proposed MX-helix at the N-terminal portal of the TM3–4 loop determine ion channel properties (Figure 1C). The integrity of this positively charged domain is important for proper membrane integration of the apolar TM3 (Sadtler et al., 2003). Neutralization of one or two basic residues resulted in translocation to the endoplasmic reticulum (ER).

Furthermore, some residues of the basic motif (^318^RRKRR^322^ in GlyRα1; ^324^RRKRK^328^ GlyRα3) are parts of a nuclear localization signal (NLS). Residues of the NLS interact with karyopherins α3/α4 and are actively involved in the nuclear import of GlyRs (Figure 1C; Melzer et al., 2010). Although, the function of GlyRs within the nucleus is unknown, an important function of nuclear import in non-neuronal tissue (Van den Eynden et al., 2009) and brain tumors has been demonstrated (Förstera et al., 2014). In glioma, a knockdown of the NLS-containing GlyRα1 reduced the self-renewal capacity of glioma formation *in vivo* and therefore impaired tumor progression.

Within the basic stretches, residues ^316^RFRRK^320^ and ^385^KK^386^ are critical for binding cytosolic G-protein subunits (Gβγ; Yevenes et al., 2006) which in turn enhance the glycine-induced chloride currents *in vitro* (Yevenes et al., 2003). It has been further estimated that the interaction of the sequences ^316^RFRRK^320^ and ^385^KK^387^ with the G-protein subunit Gβγ correlates with an allosteric interaction of the same motifs with ethanol (Yevenes et al., 2010). A peptide composed of the motif ^316^RFRRKRR^322^ was able to inhibit binding of Gβγ to the GlyRα1 intracellular loop and thus decreased the positive modulation by ethanol (Figure 1C; San Martin et al., 2012). Further determinants for ethanol binding are localized in TM2, the alternative splicing cassette within the TM3–4 loop of the α1 subunit and within the short extracellular C-terminus (Sánchez et al., 2015). Directly correlated to these data is knowledge from knock-in mice carrying K385A/K386A substitutions which show a reduced sensitivity for ethanol (Aguayo et al., 2014). K385 also plays an important role in the allosteric modulation by endocannabinoids (Yevenes and Zeilhofer, 2011). Although the GlyRα3 subunit shares sequence similarities with the GlyRα1 in terms of basic residues, GlyRα3 subunits have not been modulated by either ethanol or by Gβγ proteins. Using a chimeric approach between α1 and α3, it was demonstrated that the 15 residues alternative splice cassette of α3 and the C-terminus contains modulatory sites for Gβγ interaction in addition to the required, but not sufficient residue G254 (Sánchez et al., 2015).

## Posttranslational Modifications—Ubiquitination and Phosphorylation

Residues within the ICD of GlyRs are modulated by posttranslational modifications. Ubiquitination of postsynaptic proteins marks proteins for proteolytic degradation (Christianson and Green, 2004). Many recessive hyperekplexia mutations cause an accumulation of GlyR protein in the ER and within Golgi compartments and influence ubiquitin-mediated receptor degradation (Villmann et al., 2009b; Schaefer et al., 2015). It is proposed that ubiquitination of the GlyRα1 subunit takes place at 3 out of 10 lysine residues within the TM3–4 loop triggering receptor internalization and proteolytic degradation (Figure 1C). Proteolytic cleavage of the full-length GlyRs generates two fragments of 13 kD and 35 kD (Buttner et al., 2001). These two fragments have never been observed at the cellular surface. Processing of GlyR receptors is therefore a downstream process of ubiquitination within the endocytic degradation pathway.

GlyR subtypes are phosphorylated by protein kinases A and C (PKA and PKC; Figure 1C). Both kinases influence the maximal chloride influx and desensitization (Vaello et al., 1994; Gentet and Clements, 2002). Residue S391 within the TM3–4 loop of GlyRα1 was identified as a PKC-binding site (Ruiz-Gómez et al., 1991). Phosphorylated α1 receptors regulate channel activity and modulate the interaction with other intracellular proteins (Changeux et al., 1984). A stimulation of PKC by phorbol 12-myristate (PMA) led to an enhanced GlyR internalization rate via endocytosis. Mutation of a di-leucine motif (L314/L315) within the TM3–4 loop prevented the PMA-stimulated receptor endocytosis (Huang et al., 2007). Phosphorylation of S403 of the GlyRβ subunit reduces the affinity between the GlyRβ TM3–4 loop and gephyrin resulting in enhanced lateral diffusion of GlyRs and less synaptic GlyR levels (Specht et al., 2011).

Phosphorylation of the GlyRα3 subunit plays an important role in pain sensitization processes. PGE2 inhibits glycinergic neurotransmission via a PKA-dependent pathway (Harvey et al., 2004). The sequence Arg-Glu-Ser-Arg in the TM3–4 loop of GlyRα3 represents a strong consensus sequence for PKA. PGE2 receptors activate PKA, which in turn enhances the fraction of phosphorylated GlyRα3 via residue S346 within the PKA consensus sequence. A decrease in glycinergic signal transduction is a consequence of increased internalization of phosphorylated GlyRα3. Residue S346 is not conserved in α1 and therefore α1 lacks modulation by PKA (Harvey et al., 2004). This study clearly showed the unique role of phosphorylated GlyRα3 in spinal nociceptive processes, whereas phosphorylation of GlyRα1 controls spinal motor circuits.

Furthermore, evidence of conformational GlyR modulation by phosphorylation have been obtained in a combined approach of voltage clamp fluorometry and pharmacological measurements. The GlyRα3 S346 mutant was unable to induce conformational changes in the extracellular ligand-binding site compared with wild-type α3. These data showed for the first time that phosphorylation encompasses structural changes in the TM3–4 loop that propagate towards the ECD of the receptor (Han et al., 2013b).

SUMOylation is another type of posttranslational modification influencing receptor endocytosis and ion channel function. Although direct SUMOylation of GlyRs has never been shown, SUMOylation of kainate receptors indirectly influences GlyR endocytosis (Konopacki et al., 2011; Chamberlain et al., 2012). Recently, another kainate-induced mechanism for GlyR endocytosis has been resolved. This process involves a calcium-dependent de-SUMOylation of PKC. Activation of PKC by de-SUMOylation reduced GlyR-mediated synaptic activity concomitant to GlyR endocytosis (Sun et al., 2014). This crosstalk between excitatory and inhibitory receptors may serve to maintain the excitatory–inhibitory balance in the CNS.

## ICD Interaction with Scaffold Proteins Enables Inhibitory Synapse Formation

The best analyzed interaction between the GlyR and an intracellular binding partner is the interaction of the GlyRβ subunit with the scaffold protein gephyrin. This direct interaction involves GlyRβ residues 398–410 (Kim et al., 2006).

Gephyrin itself is a cytoplasmic protein, which consists of N-terminal G domains and C-terminal E domains (homologous to *E. coli* proteins MogA and MoeA—molybdenum cofactor biosynthetic proteins, Schwarz et al., 2001) connected by a central linker region. These domains form a hexagonal structure built up by G domain trimers and E domain dimers (Saiyed et al., 2007) anchoring GlyRs at the postsynaptic membrane (Kneussel and Betz, 2000). The binding motifs of the gephyrin E domain to GABA_A_ receptors (Maric et al., 2014) and the GlyRβ TM3–4 loop sequence ^398^FSIVGSLPRDFELS^411^ (Figure 1D) have been identified (Meyer et al., 1995). Besides its role as an anchoring protein, gephyrin undergoes interactions with polymerizing tubulin (Kirsch et al., 1991) as well as the microtubuli-associated motor proteins KIF5 and dlc1/2. These interactions are involved in anterograde and retrograde transport mechanisms of GlyRs at inhibitory synapses (Fuhrmann et al., 2002; Maas et al., 2009). Among numerous intracellular proteins bound to gephyrin, the GDP/GTP-exchange factor CB is especially interesting (Kins et al., 2000; Fritschy et al., 2008). Knockout of CB results in a region-specific loss of gephyrin in the hippocampus and gephyrin-binding GABA_A_ receptor subtypes in the forebrain of knockout mice (Papadopoulos et al., 2007, 2008). Although several attempts have been started to identify novel interaction partners of the GlyR TM-3–4 loop using yeast two hybrid screens, mostly gephyrin has been detected due to its high affinity for the GlyRβ loop. One might conclude that the affinity between other intracellular binding partners and GlyRs may be too low with respect to the sensitivity of a yeast two hybrid approach.

Using mass spectrometry, transport proteins Vps35 and neurobeachin (Nbea) and the F-bar protein syndapin I were detected as binding partners of the GlyRβ TM3–4 loop (Del Pino et al., 2011, 2014). Syndapines are important for vesicle formation at the cellular membrane, within the trans-Golgi network and the proteasome (Qualmann and Kelly, 2000; Kessels and Qualmann, 2004). Thus, the GlyRβ TM3–4 loop acts as an adapter for other intracellular binding partners involved in transport processes of receptor complexes towards the cellular membrane.

## Desensitization

**Figure 4:**
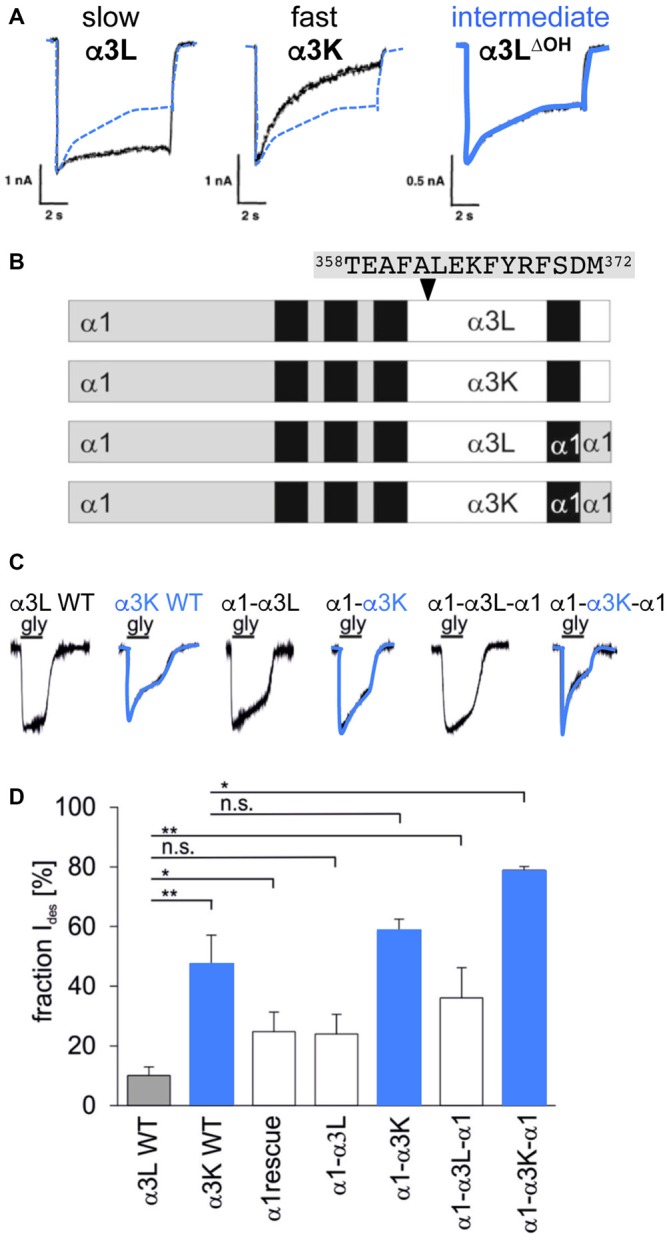
**Desensitization determined by alternative splicing cassette in GlyRα3. (A)** Desensitization of recombinant α3 glycine receptors, left—α3L almost non-desensitizing (black curve; contains alternative splicing cassette), middle—α3K fast desensitizing (black curve; without alternative splicing cassette), right α3L^ΔOH^ intermediate desensitization (shown in blue; with alternative splicing cassette but mutated hydroxylated residues α3L^ΔOH^ = α3L^T358A/Y367F/S370A^). The curve of the intermediate state is also shown in panels of α3L and α3K for comparison (dotted blue line). Note, differences in desensitizing current fractions: α3L 18%, α3K 83%, and α3L^ΔOH^ 45%, modified from Breitinger et al. (2002). **(B)** GlyRα1-α3 chimera with either the TM3–4 loop, TM4 and the C-terminus of α3L or α3K, or the TM3–4 loop only of α3L or α3K with the remaining sequence of α1. The 15 residues of the alternatively spliced segment are depicted above the scheme (positions 358–372). **(C)** Maximal glycine-evoked currents (I_max_) recorded using whole-cell configurations from HEK293 cells expressing chimeric GlyRs. All chimeras responded to saturating glycine concentrations but differed in their desensitization kinetics. Variants harboring the α3K TM3–4 loop are fast desensitizing shown by blue overlays of the appropriate current traces. **(D)** Fractions of desensitizing currents of α1α3 chimera compared to α3L (non-desensitizing) and α3K (desensitizing), blue boxes refer to chimeras containing the TM3–4 loop of α3K (modified from Meiselbach et al., 2014). n.s., not significant. Level of significance, *p < 0.05, **p < 0.01.

Desensitization is defined as the transition of the agonist-bound open channel into a closed ion channel configuration in the presence of agonist. Wild-type α1 and α3 GlyRs show very small portions of desensitizing currents. *In vitro* mutagenesis studies on the TM3–4 loop of various GlyRα subunits revealed single amino acids and grouped residues involved in the desensitization process of GlyR channels (Nikolic et al., 1998; Breitinger et al., 2009; Meiselbach et al., 2014). The human GlyRα3 carries an alternative-splicing cassette of 15 residues within the TM3–4 loop. The resulting variants α3L (including the 15 residues) and α3K (short, lacking the alternative-splicing cassette) differ significantly in their desensitization behavior (Nikolic et al., 1998). These data provided first evidences for the importance of the intracellular TM3–4 loop for ion channel desensitization (Figure 1C). The lack of this alternative-splicing cassette generated fast desensitizing currents in contrast to almost no desensitization observed for the long GlyRα3 variant (Nikolic et al., 1998). The alternative-splicing cassette of GlyRα1 subunit does not influence receptor desensitization most probably due to differences in amino acid composition compared to α3. The α3 cassette harbors three possible phosphorylation consensus sites. A substitution of residues carrying hydroxyl side chains (α3L^ΔOH^ = α3L^T358A/Y367F/S370A^) within the 15 amino acid insert generated an intermediate state of desensitization between α3L and α3K suggesting that hydroxyl groups mediate desensitization processes (Figure 4A; Breitinger et al., 2002). In a follow-up study, the secondary structure analysis of α3K and α3L suggested a stabilization of the overall spatial structure of the TM3–4 loop by the α3 splice cassette (Breitinger et al., 2009). The importance of the alternative-splicing cassette was further supported in an *in vitro* study of α1α3 chimeric proteins. The analysis of α1α3 chimera allocated that desensitization properties are transferable between GlyR subunits (Figures 4B–D; Meiselbach et al., 2014). Chimeras containing the α3 insert desensitized significantly slower than chimeras lacking the splice cassette.

The TM3–4 loop length differences between prokaryotic and eukaryotic CLRs (Tasneem et al., 2005) posed the following question: Is the TM3–4 loop essential for CLR function? Crystal structures of the prokaryotic channels ELIC and GLIC revealed both the open conformation (GLIC) and the closed channel conformation (Hilf and Dutzler, 2008, 2009; Bocquet et al., 2009). Although first studies indicated a non-desensitized GLIC in an acidic environment (Bocquet et al., 2007), GLIC desensitization became obvious at a pH lower than 5 (Gonzalez-Gutierrez and Grosman, 2010; Parikh et al., 2011). These data again argue for subtype-specific regulatory elements of desensitization within the CLR superfamily. An exchange of the whole TM3–4 loop of various CLRs (5HT_3_ and GABA_C_ receptor) with the ICD of GLIC (SQPARAA) did not lead to changes in the macroscopic electrophysiological properties of the chimeric ion channels (Jansen et al., 2008; Papke and Grosman, 2014). In a recent study, the full-length loop of GlyRα1 was either replaced completely by the prokaryotic heptapeptide (i), or (ii) basic stretches ^318^RRKRR and ^393^KKIDK close to TM3 and 4 have been left intact carrying the heptapeptide in between (GlyRα1-GLIC(+)bm). (iii) A third construct contained a short TM3–4 loop only composed of both basic stretches (GlyRα1-ΔTM3–4(+)bm; Figure 3C). The pure heptapeptide between TM3 and TM4 resulted in intracellular aggregation, lack of surface receptors and non-functionality. Constructs GlyRα1-GLIC(+)bm (ii) and GlyRα1-ΔTM3–4(+)bm (iii) were able to form functional ion channels that differed significantly in their desensitization behavior. The presence of both basic stretches resulted in a fast transition of GlyRα1 channels into a closed conformation. The insertion of SQPARAA between both basic motifs (GlyRα1-GLIC(+)bm) decreased the desensitizing current significantly in comparison to wild-type GlyRα1 (Figure 3D). Thus, the sequence between both basic stretches determines the desensitization behavior of GlyRα1 (Langlhofer et al., 2015). The introduction of the prokaryotic heptapeptide at another position within the GlyRα1 TM3–4 loop between residues Q310 and K385 depicted also differences on the fraction of desensitizing currents (Papke and Grosman, 2014). The common conclusion from studies concerning the length of TM3–4 loop and the determination of desensitization rates revealed that separation of both basic stretches at the N- and C-terminal end of the TM3–4 loop represent a critical determinant of ion channel functionality.

To complete the knowledge on desensitization determined by the GlyR ICD, the human mutation P250T needs to be mentioned. This mutant localized in the M1-M2 loop is associated with very fast desensitization. The original proline introduces conformational rigidity to the short M1-M2 linker. The given higher flexibility by the introduced threonine allows TM2 rearrangements resulting in fast ion channel closure. Thus, fast desensitization underlies the pathology of patients carrying P250T and in turn contributes to enhanced muscle tone delineating a major clinical feature in startle disease patients (Saul et al., 1999; Breitinger et al., 2001). Further support for a key role of the M1-M2 loop in desensitization derives from a recent study on the identification of the desensitization gate in CLRs. The TM1–2 loop interacts with the internal end of TM3 determining the desensitization gate. An exchange of GlyR residues with residues from the GABA_C_ ρ1 subunit elicited the intracellular end of TM3 as the key component for desensitization (Gielen et al., 2015). Further hints for an association of enhanced desensitization and disease were given by studies of the nAChR. The enhanced desensitization of presynaptic nAChRs at GABAergic terminals generates lower inhibitory input at dopaminergic neurons and concomitantly enhanced activity of the dopaminergic rewards system (Mansvelder et al., 2002). An enhanced desensitization rate of nAChRs has also been described to underlie a special form of frontal lobe epilepsy (Bertrand et al., 2002).

## Conclusions and Outlook

The ICD of the glycine receptor harbors subdomains important for trafficking and functionality of the inhibitory GlyR. Basic residues are crucial determinants in both processes. Since trafficking is a prerequisite for functional modulation, the basic domains represent key regulators of this receptor family. This is further supported by their involvement in binding of Gβγ proteins and ethanol.

Studies on chimeric proteins have helped us to understand the functional role of the TM3–4 loop. Lack of this large intracellular loop does not lead to non-function, rather to a disruption of ion channel modulation. Except for the cytoplasmic portals that are proposed to resemble an α-helical structure, the TM3–4 loop is suggested to be unfolded. Unfolding might represent an advantage for the interaction with intracellular proteins important for regulation of receptor recruitment to synaptic sites, ion channel function, and finally degradation initiation. Further research is required to enhance our knowledge on other so far non-identified interactions partners modulating synaptic strength and fine-tuning of GlyR function depending on the surrounding neuronal network.

## Author Contributions

GL and CV wrote the manuscript.

## Conflict of Interest Statement

The authors declare that the research was conducted in the absence of any commercial or financial relationships that could be construed as a potential conflict of interest.

## Abbreviations

CLRs: Cys-loop receptors
ECD: extracellular domain
ICD: intracellular domain
TM: transmembrane
GlyR: glycine receptor
wt: wild-type.
